# Identification of Differentially Expressed Genes in Cervical Cancer Patients by Comparative Transcriptome Analysis

**DOI:** 10.1155/2021/8810074

**Published:** 2021-03-19

**Authors:** Annapurna S. D., Deepthi Pasumarthi, Akbar Pasha, Ravinder Doneti, Sheela B., Mahendran Botlagunta, Vijaya Lakshmi B., Smita C. Pawar

**Affiliations:** ^1^Department of Genetics, University College of Science, Osmania University, Hyderabad, 500 007 Telangana, India; ^2^MNJ Institute of Oncology & Regional Cancer Center, Red Hills, Hyderabad, 500004 Telangana, India; ^3^Department of Indo American Cancer Research Foundation, Basavatarakam Indo American Cancer Hospital and Research Institute, Banjara Hills, Hyderabad, Telangana, India; ^4^Institute of Genetics and Hospital for Genetic Diseases, Osmania University, Begumpet, Hyderabad, Telangana, India

## Abstract

Cervical cancer is one of the most malignant reproductive diseases seen in women worldwide. The identification of dysregulated genes in clinical samples of cervical cancer may pave the way for development of better prognostic markers and therapeutic targets. To identify the dysregulated genes (DEGs), we have retrospectively collected 10 biopsies, seven from cervical cancer patients and three from normal subjects who underwent a hysterectomy. Total RNA isolated from biopsies was subjected to microarray analysis using the human Clariom D Affymetrix platform. Based on the results of principal component analysis (PCA), only eight samples are qualified for further studies; GO and KEGG were used to identify the key genes and were compared with TCGA and GEO datasets. Identified genes were further validated by quantitative real-time PCR and receiver operating characteristic (ROC) curves, and the highest Youden index was calculated in order to evaluate cutoff points (COPs) that allowed distinguishing of tissue samples of cervical squamous carcinoma patients from those of healthy individuals. By comparative microarray analysis, a total of 108 genes common across the six patients' samples were chosen; among these, 78 genes were upregulated and 26 genes were downregulated. The key genes identified were SPP1, LYN, ARRB2, COL6A3, FOXM1, CCL21, TTK, and MELK. Based on their relative expression, the genes were ordered as follows: TTK > ARRB2 > SPP1 > FOXM1 > LYN > MELK > CCL21 > COL6A3; this generated data is in sync with the TCGA datasets, except for ARRB2. Protein-protein interaction network analysis revealed that TTK and MELK are closely associated with SMC4, AURKA, PLK4, and KIF18A. The candidate genes SPP1, FOXM1, LYN, COL6A3, CCL21, TTK and MELK at mRNA level, emerge as promising candidate markers for cervical cancer prognosis and also emerge as potential therapeutic drug targets.

## 1. Introduction

Cervical cancer is the most commonly diagnosed gynaecological cancer after breast cancer worldwide [1–4]. In spite of the effective screening programs and vaccination, incidence and mortality rates are increasing alarmingly. Nearly 570,000 new cases are diagnosed with the disease resulting in the death of about 311,000 cases per year in well-developed countries [3]. As per global mortality rates, India alone accounts for one-fourth part of the cervical cancer deaths [2]. The incidence of the disease is very high in India, nearly 122,844 women are diagnosed with the disease and around 67,477 deaths are reported annually [5]. It is a renowned fact that human papillomavirus (HPV) infection plays a prominent role in the pathogenesis of cervical cancer and more than 70 percent of cervical cancer cases can be accredited to two types of virus, i.e., HPV-16 and HPV-18 [6]. Women infected with human papillomavirus (HPV) progress to precancerous stages LSIL (low-grade squamous intraepithelial lesions/CIN I) and HSIL (high-grade intraepithelial lesions or CIN II/CIN III), and these high-grade lesions further lead to invasive cervical cancer over a period of time [6, 7]. Oncogenic HPV infection alone is insufficient to cause malignancy; subsequently, other related genetic factors like smoking, long-term usage of oral contraceptives, high parity, sexual behaviour, unwillingness to undergo screening, and improper hygienic conditions contribute significantly [6, 8, 9]. Deciphering the key concepts involved in the transformation of malignancy and advancement of the disease, comprehending the underlying molecular mechanisms and detecting cancer at an early stage might reduce the mortality rate and would also provide better prognosis. Very limited studies have been carried out to understand the initiation, progression, and pathology of this disease in India; therefore, this study was performed to provide a comprehensive transcriptome analysis of cervical cancer patients of Indian origin. Molecular signatures for better prognosis and potential therapeutic targets from this data analysis were identified and further confirmed by comparing it to the TCGA datasets.

## 2. Materials and Methods

### 2.1. Sample Collection

A total of 10 tissue samples, 7 biopsies from cervical cancer patients (squamous cell carcinoma) and 3 from the normal subjects, were collected from the Mehdi Nawaz Jung (MNJ) Cancer Hospital and CC Shroff Maternity Hospital, Hyderabad. Normal cervical tissues (nontumor samples) were obtained from the women who underwent hysterectomy for other gynaecological-related problems. The institutional ethical committee review board of Osmania University and MNJ Cancer Hospital has approved the study.

### 2.2. DNA Isolation and Detection of HPV

DNA was isolated from the tumor and nontumor (controls) biopsy samples using the QIAamp DNA mini kit (Cat: 51104), and the quality was determined by biospectrophotometer (Eppendorf). The DNA isolated was subjected to beta-globin (housekeeping gene) PCR using specific primers to check the quality of DNA. The GP5+/GP6+ primers [10], which amplify the HPV DNA by binding to the L1 region of the HPV genome, were used to detect the human papillomavirus (HPV) positivity in the DNA samples of both tumors and nontumor samples. The primer sequences have been given in Table 1.

### 2.3. RNA Isolation

Total RNA was isolated from cervical cancer (tumor) and normal cervix (nontumor) tissues by using the RNeasy Plus universal kit (Qiagen, Cat No: 73404). The quality of RNA was affirmed by using biospectrophotometer (Eppendorf), and RIN (RNA integration number) values were assessed using Agilent 2100 bioanalyzer. To check the integrity of 28s and 18s rRNA, the total RNA was run on formaldehyde agarose gel and the degraded samples were excluded from the study.

### 2.4. Affymetrix Microarray Hybridization Analysis

A total of 10 high-quality RNA samples were labelled and hybridized on human Clariom D gene chips, as per the manufacturer's protocol (Affymetrix, Santa Clara, CA, USA # 902922). Briefly, double-stranded complementary DNA (cDNA) and complementary RNA (cRNA) were synthesized from total RNA, and then, biotinylated cDNA was hybridized onto the human transcriptome array 2.0 for 16 hours in an Affymetrix GeneChip 645 hybridization oven at 45°C. The arrays were stained by using GeneChip Fluidics Station 450. Later, the chip was scanned with GeneChip™ scanner 3000 [11]. The fluorescent signals of the array were obtained as DAT files. Raw data of ARR and DAT image files contain pixel intensity values. Affymetrix GeneChip Command Console (AGCC) software is used for converting the raw data of ARR and DAT image files into intensity data (.CEL files and .CHP files). Affymetrix Clariom D .CEL files were normalized to produce probe-level signal expression values (.CHP files) by using Expression Console (EC) software (version 1.4.1). The CHP files were transferred to transcriptome analysis console (TAC) software (version 4.0.2) and analyzed the expression pattern of the genes, exons, splice variants, and the related pathways involved in the cervical cancer progression [12].

The microarray data have been submitted to the GEO database with accession number GSE127265 with the link https://www.ncbi.nlm.nih.gov/geo/query/acc.cgi?acc=GSE127265.

### 2.5. Creation of Venn Diagram Using Python

To generate the Venn diagrams for common up and downregulated genes from 6 patients, we used Pyvenn (https://github.com/tctianchi/pyvenn) and Pandas packages (https://pypi.org/project/pandas) of python.

### 2.6. DEGs Validation by Quantitative PCR (qPCR)

Eight significant target genes obtained from transcriptome data were selected to perform the quantitative PCR. Primer sequences of selected DEGs are given in Table 1. In brief, 1 *μ*g of total RNA from each sample (tumor and nontumor) was reverse-transcribed by iScript™ cDNA synthesis kit (Bio-Rad, Cat#1708891). From that, 50 ng of reverse-transcribed RNA (1 *μ*l) was used to perform the expression analysis of selected genes by using SYBR Green Master Mix (KAPA SYBR® FAST (2X) Universal, Cat no: KK4601) in Agilent AriaMx real-time PCR detection system, with a final volume of 20 *μ*l reaction in each well. The protocol consists of 40 cycles at 95°C for 10 minutes (hot start), 95°C for 15 seconds (melting), and 60°C for 30-60 sec (annealing and extension), as recommended by the manufacturer's instructions. All the samples were processed in triplicates, and beta-actin gene was used as an endogenous control for reference. The results were normalized with endogenous control by using the Livak method (2^−ΔΔCt^) [13]. Statistical analysis for qPCR was performed in GraphPad Prism 6 (GraphPad Software Inc., San Diego, CA), mean ± standard error (SEM), and mean ± standard deviation (SMD) where all the values were calculated by Student's *t*-test with a significant *p* value < 0.05.

### 2.7. Receiver Operating Characteristic (ROC) Curves

Receiver operating characteristic (ROC) curve displays the discriminatory accuracy of the marker for distinguishing between two groups. The ΔCT values of each sample were used to plot receiver operating characteristic (ROC) curve. ROC is a plot of the sensitivity (true positive rate) vs. 1 − specificity (false positive rate) and is used for estimating possible threshold values of all the identified markers. The area under the ROC curve (AUC) value of the DEGs defines the usefulness of markers with respect to its ability to separate the two different groups of cervical tissue biopsies. The highest Youden index was calculated by using the ROC curve; the *Y*-index is associated with optimal threshold cutoff point (COP) for the DEGs. The COP values indicate increased or decreased expression levels of DEGs in cancer and normal samples. All the plots were obtained by using R-Studio version 3.6.3.

### 2.8. Gene Ontology (GO) and KEGG Functional Enrichment Analysis

To determine the significantly implicated functional genes and biological pathways of selected DEGs, enrichment analysis was performed from the publicly available bioinformatics online database DAVID (The Database for Annotation, Visualization and Integrated Discovery) (Version 6.8, https://david.ncifcrf.gov), which aids in analyzing the GO and KEGG pathways of dysregulated genes. Gene Ontology (GO) is a standard recognized classification system for defining biological functions (BF), molecular functions (MF), and cellular components (CC) of differentially expressed genes, and KEGG (Kyoto Encyclopedia of Genes and Genomes) pathway enrichment analysis was used to determine the significant signaling pathways with significant *p* value < 0.05.

### 2.9. Gene Expression Data Retrieved from GEO and GEPIA

The microarray gene expression profiles of GSE63514 and GSE9750 were obtained from the publicly available NCBI-Gene Expression Omnibus (GEO) database of cervical cancer (http://www.ncbi.nlm.nih.gov/geo). Gene expression profiling interactive analysis (GEPIA) web tool was used to analyze the box plots of RNA sequencing expression data from cervical cancer squamous cell carcinoma (CESC) of TCGA dataset (306 tumor and 13 nontumor samples) and GTEx projects [14]. These two data mining sites were used to cross-validate the expression of DEGs in cervical cancer tissues in comparison to the normal tissues.

### 2.10. Construction of Protein-Protein Interaction (PPI) Network

The differentially expressed genes (DEGs) obtained from microarray analysis were analyzed by using a bioinformatics tool STRING (Search Tool for the Retrieval of Interacting Genes/Proteins) Cytoscape version 3.6.1 [15]. It predicts the potential interaction between genes at the protein level; a combined score greater than 0.4 was used to construct the PPI network of the proteins. In this composite network, each node represents a specific protein and each edge (line) represents the interaction among the proteins.

## 3. CytoHubba

CytoHubba plugin Cytoscape explores the significant hub genes from the biological networks employed by eleven node ranking methods which include local-based methods (degree, edge percolated component, maximum neighborhood component (MNC), density of maximum neighborhood component (DMNC), and maximal clique centrality) and global-based methods (bottleneck, eccentricity, closeness, radiality, betweenness, and stress). It uses ranking features to rank different nodes in a network, and based on their values, hub genes are reported. Maximum clique centrality (MCC) is a better scoring method to identify essential nodes from the network [16]. Based on the scoring, CytoHubba finally selects top 10 proteins from the given network.

## 4. Results

### 4.1. Identification of Gene Signatures Using Microarray Data in Cervical Cancer

**Figure 1:**
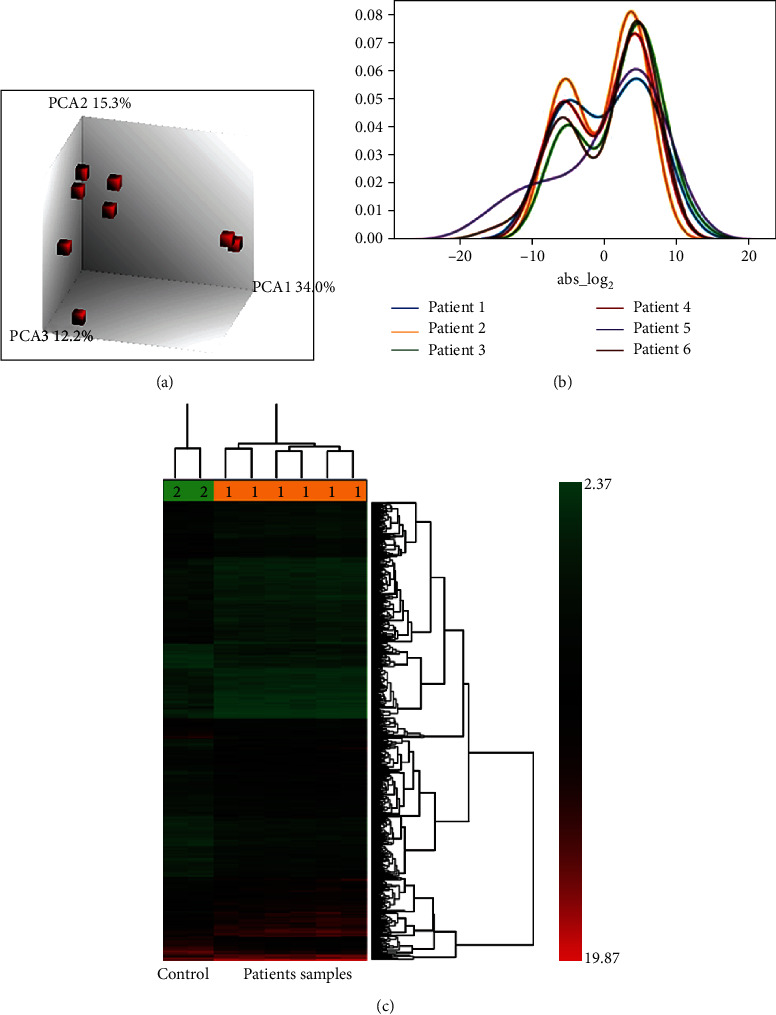
Identification of gene signatures using microarray data analysis in cervical cancer. (a) Principal component analysis (PCA) of transcriptome data. Three-dimensional scatter plot represents the gene expression patterns between patients' and control samples. (b) Hierarchical clustering displays differentially expressed genes in cervical squamous cell carcinoma. Red color indicates a high-level expression of genes (fold change > 2), and green color indicates the low-level expression of genes (fold change < 2) with *p* value < 0.05. (c) Distance plot shows frequencies of up- and downregulated genes in patients' samples.

This study intended to identify the differential gene expression pattern in cervical cancer patients of Indian origin. Cervix tissue biopsies were collected, and HPV infection was determined by HPV consensus PCR (Figure S1). Only HPV-positive samples were selected for total RNA isolation, and quality of the RNA was verified by 1.2% formaldehyde gel. Total RNA was subjected to microarray analysis using the human Clariom™ D chip array (Affymetrix, Santa Clara, CA). The gene expression data analysis was performed by two sequential steps, (a) preprocessing (normalization) and (b) descriptive statistics. For preprocessing, we used SST-RMA (signal space transformation-robust multichip analysis) algorithm to normalize the signal intensity of the expression data. After normalization, data is subjected to PCA (principal component analysis) for monitoring the distribution of samples. Based on the PCA, two samples (1 control and 1 diseased with IDs GSM3633379 and GSM3633376) were found to be outliers; hence, not included in further analysis, and the remaining samples were distributed into two groups, six patients' samples in one group and 2 controls in another group (Figure 1(a)). Hierarchical clustering is a descriptive statistical method employed to identify the differential expressed genes with a fold change (≥2 and ≤-2) and ANOVA *p* value of ≤0.05. The results revealed clustering of that data into two, namely, clusters A and B representing control and patients (Figure 1(b)). In the figure, the red color represents upregulated and green color represents downregulated genes. A total of 15,325 genes were differentially expressed in patients when compared to controls, and six patients showed 1267, 3463, 2726, 2741, 2102, and 3026 transcripts, respectively. Subsequently, we plotted a distance plot, to find whether transcripts (up and down) were distributed equally among the patients or not. The frequency of upregulated genes in all patients is higher than downregulated genes (Figure 1(c)). Patient 1 showed 892 up (coding-147) and 375 down (coding-59), patient 2 showed 1802 up(coding-155) and 1661 down (coding-337), patient 3 showed 1526 up (coding-149) and 1200 down (coding-201), patient 4 showed 1475 up (coding-143) and 1266 down (coding-208), patient 5 showed 1254 up (coding-131) and 848 down (coding-157), and patient 6 showed 1748 up (coding-209) and 1278 down (coding-214) dysregulated genes.

### 4.2. Identification and Validation of Common Differentially Expressed Genes (DEGs) in Cervical Cancer Patients

**Figure 2:**
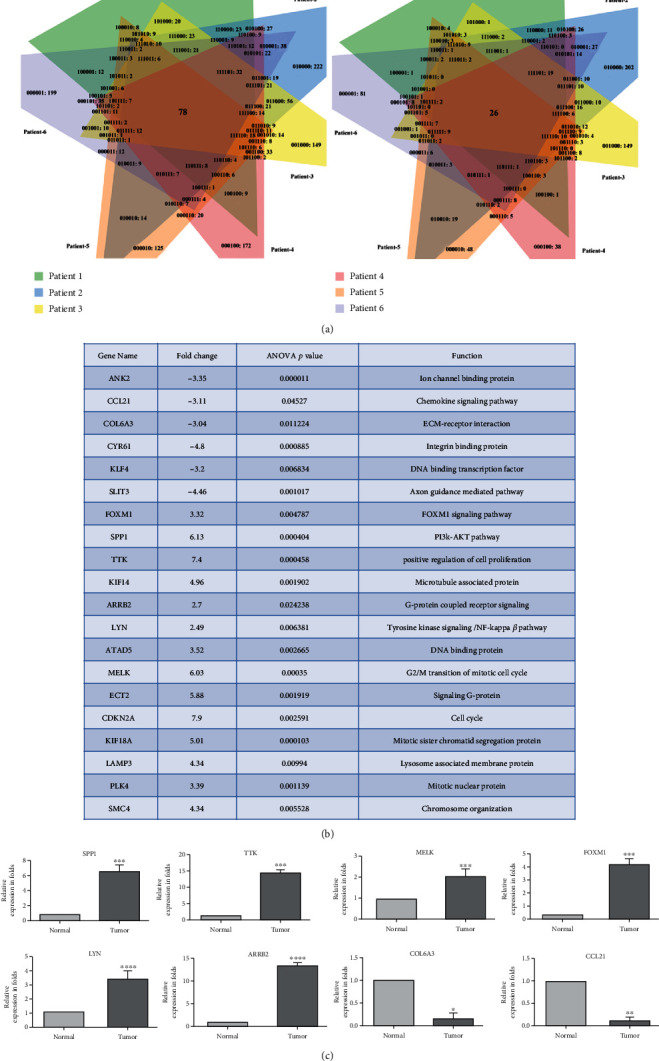
Identification of common deregulated genes in cervical cancer patients. (a) Venn diagram representing the total number of common upregulated (left panel) and downregulated (right panel) DEGs in patients' samples. (b) A set of key genes involved in molecular signaling pathway based on the GO and KEGG pathway analysis. (c) Relative expression of dysregulated genes validated by qPCR. Statistical analysis revealed that the difference in expression between normal and tumor was significant (*p* ≤ 0.05).

To identify the common genes involved in the progression of cervical cancer across patients, we performed a comparative analysis using Venn diagram software. The results showed that a total of 104 common DEGs were identified, of which 78 are upregulated (logFC > 2) and 26 are downregulated (logFC < -2) (Figure 2(a)). To identify the cellular pathways associated with the progression of cancer, these 104 common genes were subjected to GO functional and KEGG pathway enrichment analysis by using DAVID database. Of these, 20 genes are involved in chemokine signaling, tyrosine kinase signaling, cell division, and cell-cell signaling pathways which are listed in the table (Figure 2(b)). In order to validate the microarray findings, eight DEGs were selected based on the involvement of cancerous pathways in other cancers and also in cervical cancer, but very limited studies have been reported in Indian population: SPP1 (PI3K-Akt signaling pathway-hsa0415) [17], TTK (cell cycle-hsa04110) [18], MELK (cell cycle-hsa04110) [19], FOXM1 (cellular senescence-hsa04218) [20, 21], LYN (viral carcinogenesis-hsa05203) [22], ARRB2 (MAPK signaling pathway-hsa04010) [23], COL6A3 (ECM-receptor interaction) [24], and CCL21 (chemokine signaling pathway-hsa04062) [25]. These 8 genes (SPP1, TTK, MELK, FOXM1, LYN, ARRB2, COL6A3, and CCL21) were further validated in more numbers of cancer tissue samples by quantitative real-time PCR (qRT-PCR). Among these, TTK gene showed significant upregulation followed by ARRB2 (TTK > ARRB2 > SPP1 > FOXM1 > LYN > MELK). CCL21 was found to be significantly downregulated as compared to COL6A3 as shown in (Figure 2(c)), and all these genes behaved similarly as seen in the microarray data and our study is in accordance with other published reports of cervical cancer.

### 4.3. Receiver Operating Characteristic (ROC) Curves Analysis

**Figure 3:**
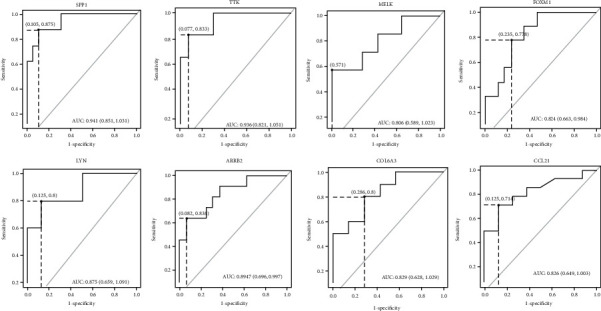
ROC curve for DEGs based on the RT-qPCR data. The figure represents a plot of the sensitivity (true positive rate) vs. 1 − specificity (false positive rate) for all the ΔCT values. The AUC values indicate that the two groups may be distinguished by expression analysis of these markers. The point on the dotted line shows the highest Youden (*Y*) indices associated with the COP. The resolute values of AUC, *Y*, and COP for the examined DEGs are listed in Table 2.

The ROC curves were graphically plotted from the data derived by RT-qPCR using the ΔCT values, and AUC was determined. The AUC (Figure 3) and COP values with the highest Youden indices for all the represented upregulated and downregulated DEGs have been listed in Table 2. The ΔCT values under the COP (upregulated) of SPP1, TTK, MELK, FOXM1, LYN, and ARRB2 and the ΔCT over the COP (downregulated) of COL6A3 and CCL21 were considered to be positive for the malignancy in the test group (cervical squamous tissue biopsies) and were statistically significant at *p* < 0.05.

### 4.4. Data Mining of Selected DEGs in Cervical Carcinoma

**Figure 4:**
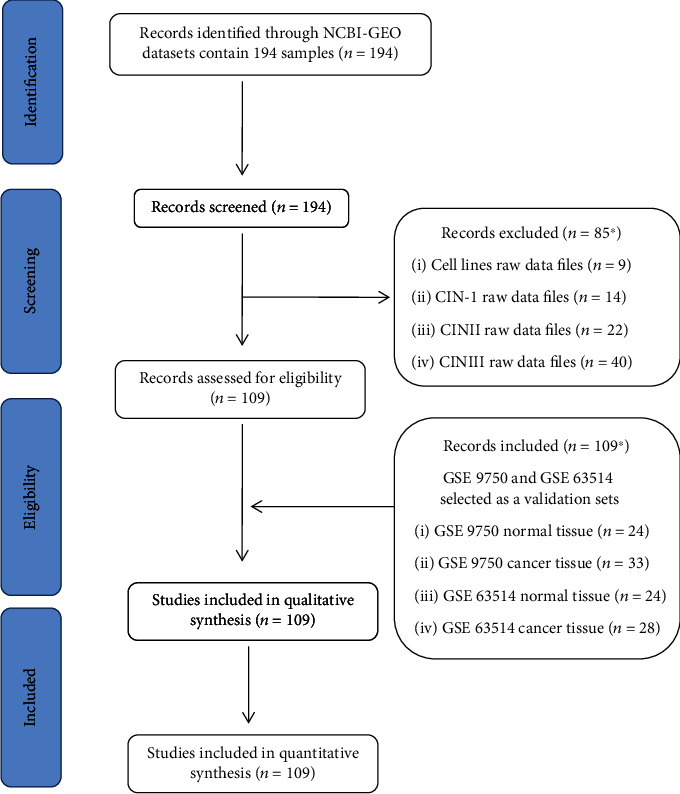
Schematic work flow (with inclusion and exclusion criteria) for GEO datasets analysis.

**Figure 5:**
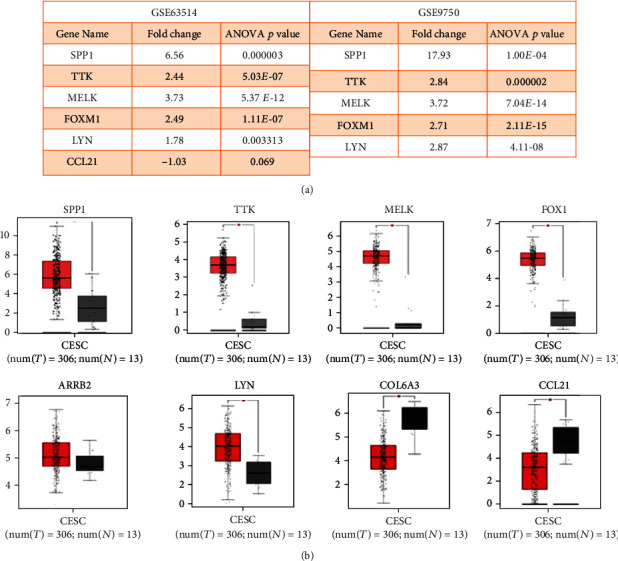
Data mining based on GEO and TCGA datasets. (a) Gene expression profile of selected DEGS in GEO datasets GSE63514 and GSE9750. (b) Box plot representing relative expression patterns of dysregulated genes in cervical squamous cell carcinoma (CESC) using the TCGA database.

To determine the clinical significance of selected DEGs in patients of cervical carcinoma, we performed data mining using the NCBI-GEO (Gene Expression Omnibus) and TCGA (The Cancer Genome Atlas) databases. NCBI-GEO datasets' raw data files were analyzed by using Expression Console (EC) and Transcriptome Analysis Console (TAC) software provided by the Affymetrix, Santa Clara, CA, USA. In brief, Expression Console (EC) software is used to convert the raw data files (.CEL files) in to probe-level signal expression values (.CHP files). The Transcriptome Analysis Console (TAC) software is used to analyze these signal expression values (.CHP files) for downstream analysis of all library files in Affymetrix platforms. The gene expression profile from the GSE63514 dataset consists of 28 cancerous and 24 normal cervical tissues, and GSE9750 dataset consists of 33 cancers and 24 normal cervical tissues; the gene chip platforms of both these datasets were Affymetrix HG-U133_Plus_2.0 and Affymetrix HG-U133A array. The inclusion criteria of selecting the datasets was that the data obtained from normal tissue and cancer tissues samples analyzed on Affymetrix platform and the raw data files from cell lines and early lesions/CIN cases were excluded as the study is aimed at identifying the dysregulated genes between normal and cancerous tissues. Schematic work flow (with inclusion and exclusion criteria) for the GEO datasets analysis is shown in Figure 4. Both GEO datasets' CEL raw files were analyzed using Expression Console and TAC software provided by the Affymetrix (Figure 5). Gene expression profile of selected DEGS in the GSE63514 and GSE9750 GEO datasets is shown in Figure 5(a). GEPIA is a web-based tool for cancer and normal gene expression profiling based on TCGA database. The interactive analysis tool was applied to confirm the expression level of the eight DEGs (SPP1, TTK, MELK, FOXM1, LYN, ARRB2, COL6A3, and CCL21) in cancer and normal tissues (Figure 5(b)). The gene expression profiles of all genes were significant except for ARRB2.

### 4.5. Protein-Protein Interaction Network of DEGs and CytoHubba

**Figure 6:**
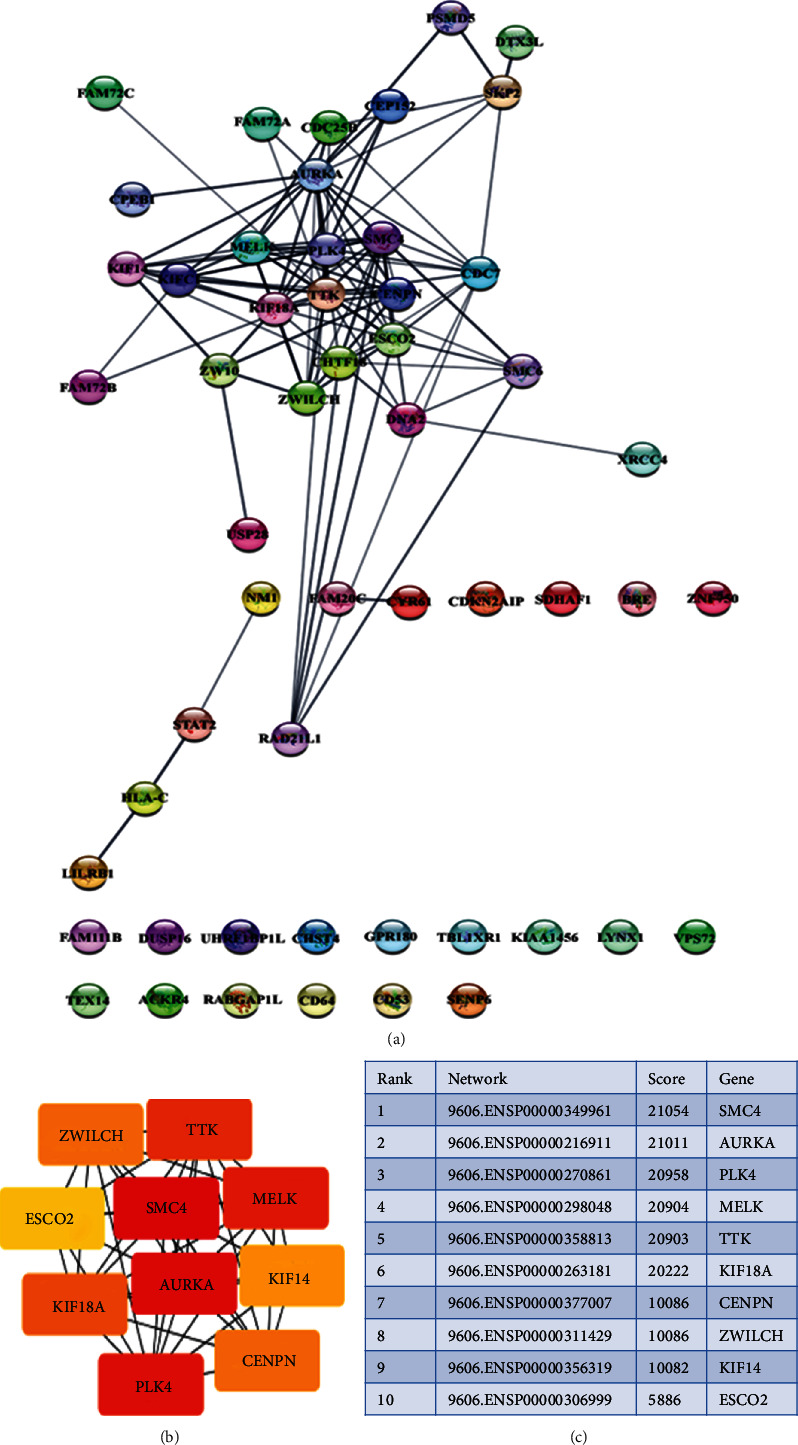
PPI network and CytoHubba. (a) STRING creates PPI network of dysregulated genes; each circle represents the node (gene). (b, c) Based on the ranking of the highly interconnected dysregulated top 10 hub proteins predicted by CytoHubba.

PPI network of dysregulated genes (78 upregulated and 26 downregulated) was constructed in the STRING with the help of Cytoscape. The resultant network has 53 nodes and 106 edges (Figure 6(a)). CytoHubba plugin selects the top 10 highest scored genes from the network by using the maximum clique centrality (MCC) scoring method (Figure 6(c)). By comparing the 8 DEGS microarray data with high scoring CytoHubba genes, it was found that only TTK and MELK proteins were interacting with other genes (Figure 6(b)). Thus, the results suggest that analyzing and validating these two genes at the protein level might be useful for therapeutic applications of cervical cancer.

## 5. Discussion

Development of cervical cancer is a complex process; persistent infection of human papillomavirus (HPV) is a prerequisite for cervical cancer and its precursor lesions [26] along with HPV; genetic and epigenetic factors also play a crucial role in pathogenesis. Microarray has been extensively used to study the genetic alterations in cancer and to identify the disease-specific prognostic biomarkers and therapeutic targets; therefore, we performed microarray-based transcriptome analysis to identify the dysregulated genes in cervical cancer.

This study revealed the 108 common genes were differentially expressed (78 upregulated and 23 downregulated genes) between patients' samples compared to the normal cervix. Based on the results obtained from the data mining and GO and KEGG pathway enrichment analysis [27], which are widely used for the selection of essential genes, we selected further a total of eight genes for experimental validation by using real-time PCR assay. The selected genes for the analysis are SPP1, TTK, MELK, FOXM1, LYN, ARRB2, CCL21, and COL6A3, and apart from CCL21 and COL6A3, all other genes were upregulated in the clinical samples. The clinicopathological significance of selected genes was analyzed by ROC curves, and the inferences of AUC for diagnostic ability of selected DEGs were specified. All the selected DEGs, both the upregulated and downregulated, showed significant investigative value for differentiating between healthy controls and patients (Figure 3, Table 2). The Youden index for the ROC curve for all the DEGs can be used as the ideal diagnostic cutoff value.

It is clear from our study that SPP1 (secreted phosphoprotein 1), also called as osteopontin, is highly upregulated in all the tested cancer samples and also has been reported in other population studies; hence, SPP1 emerges as a potential prognostic biomarker for cervical cancer screening in women [28, 29]. Aberrant activation of OPN expression has been reported in gastric, colon, renal, breast, oesophageal, and endometrial cancers [30, 31]. TTK is a dual-specificity protein kinase, and the dysregulated expression of this protein has shown to be involved in cell proliferation in multiple cancers. Upregulation of TTK has shown to be associated with malignant transformation of cervical cancer [18, 32, 33]. Maternal embryonic leucine zipper kinase (MELK) is a serine/threonine-protein kinase, which plays an important role in embryogenesis and cell cycle control. It is overexpressed in malignant tumors including hepatocellular carcinoma, breast cancer, cervical cancer, and ovarian cancer [19, 34–36]. Forkhead box M1 (FOXM1), a known driver of tumorigenesis [37], plays a key role in a wide variety of cellular processes such as cell cycle regulation (G_2_ to M progression), angiogenesis, cell differentiation, cellular senescence, and epithelial-mesenchymal transition (EMT). In the majority of cancers, overexpression of FOXM1 induces the progression of the disease [21, 37, 38]. LYN is a tyrosine kinase which is deregulated in a variety of cancers like breast, prostate, melanoma, and cervix [39–42]. *β*-Arrestin 2 (ARRB2) belongs to the arrestin family, which helps to modulate the desensitization and trafficking of G protein-coupled receptors (GPCRs). It regulates cell proliferation and promotes cell invasion and migration in renal cancer [43].

Collagen alpha-3(VI) chain (COL6A3) is associated with cell anchoring and helps in microfibril formation [44]. It forms a filamentous network with collagen types I and III and ECM remodeling to create a tumor microenvironment. A recent study by Huang et al. (2018) showed significantly upregulated expression of COL6A3 in bladder cancer. They suggested that COL6A3 may regulate the levels of EMT-related proteins to enable cell migration and metastasis [45]. Reports on gastric cancer and colorectal cancer also showed high expression of COL6A3 [24, 46], but interestingly in our study, COL6A3 has shown lower expression and this downregulation might be due to mucinous nature of the cervical tissue. CC chemokine ligand (CCL21) plays an important role in homing of immune cells, peripheral tolerance, and development and function of T regulatory cells. CCL21 mediates its activity through binding to its receptor CCR7. Various studies have shown that elevated levels of CCL21/CCR7 lead to migration and proliferation of cancerous cells [47, 48], although decreased expression of CCL21 was reported in human colorectal adenocarcinoma [49]. Downregulation of CCL21 is attributed to upregulated expression of SPP1 in cervical cancer tissue [50]. Further, we validated these DEGs by using the TCGA database. Overall, our results show concordance between the qPCR data and TCGA data.

## 6. Conclusion

We have identified a panel of genes SPP1, MELK, TTK, ARRB2, FOXM1, LYN, CCL21, and COL6A3, which were highly dysregulated in cervical squamous cell carcinoma in comparison to the normal cervical epithelium through microarray analysis, and further gene expression was validated using qPCR and analyzed by receiver operating characteristic (ROC) curves. Some of these aberrantly dysregulated genes were previously reported in other cancers and also in cervical cancer, but very limited studies have been reported in Indian population. Among these, SPP1 and CCl21 were found to be potent secretary molecules contributing to the progression of cervical cancer; thus, the current study projects these genes as potential therapeutic targets in general for cervical cancer and in specific for the Indian population. These genes need to be further validated in larger cohorts of Indian populations to establish them as potential diagnostic and prognostic markers, as well as prospective therapeutic targets for cervical cancer.

## Figures and Tables

**Table 1:** Primer sequences and amplicon sizes of selected genes used in the real-time qPCR reaction.

Genes	Forward primer	Reverse primer	Amplicon sizes (bp)
SPP1	CGAGGTGATAGTGTGGTTTATGG	GCACCATTCAACTCCTCGCTTTC	128
TTK	CCGAGATTTGGTTGTGCCTGGA	CATCTGACACCAGAGGTTCCTTG	110
MELK	TCCTGTGGACAAGCCAGTGCTA	GGGAGTAGCAGCACCTGTTGAT	153
FOXM1	GGAGCAGCGACAGGTTAAGG	GTTGATGGCGAATTGTATCATGG	115
LYN	GCTGGATTTCCTGAAGAGCGATG	CGGTGAATGTAGTTCTTCCGCTC	117
ARRB2	ACTGGACCCTCTCTTGCTGA	CTTTTCACTGTCCCCTTCCA	122
COL6A3	CCATCCGAGACTTCATTGCT	CCCTTTTTGTTGGATGGGTA	132
CCL21	AGCAGGAACCAAGCTTAGGCTG	GGTGTCTTGTCCAGATGCTGCA	133
Beta-actin	CACCATTGGCAATGAGCGGTTC	AGGTCTTTGCGGATGTCCACGT	135
GP5+/GP6+	TTTGTTACTGTGGTAGATACTAC	GAAAAATAAACTGTAAATCATATTC	150

**Table 2:** Statistical analysis based on the ΔCT values of the control vs. the patient group of DEGs.

Genes	AUC	*Y*-index	COP (ΔCT)	*p* value	95% confidence interval
SPP1	0.941	0.769	8.82	0.0003	0.851 to 1.031
TTK	0.936	0.756	9.22	0.002	0.821 to 1.051
MELK	0.806	0.571	9.96	0.025	0.589 to 1.023
FOXM1	0.824	0.542	9.56	0.007	0.663 to 0.984
LYN	0.875	0.675	10.79	0.020	0.659 to 1.091
ARRB2	0.847	0.573	7.94	0.003	0.696 to 0.997
COL6A3	0.829	0.514	3.28	0.024	0.628 to 1.029
CCL21	0.826	0.589	8.86	0.012	0.649 to 1.003

## Data Availability

Microarray data have been submitted to the GEO database with accession number GSE127265.

## Supplementary Materials

Supplementary Materials Figure S1: HPV DNA amplified by using Gp5+/Gp6 primers; lane 1 is 100 bp marker, lane 2 is control (normal sample), lane 3 is HPV-positive control, lanes 4-7 are patients' samples with a product length of 150 bp, and lane 8 is NTC (nontemplate control).
