# Supplementary figures and images for: Identification of Differentially Expressed Genes in Cervical Cancer Patients by Comparative Transcriptome Analysis
Source: Biomed Res Int. 2021 Mar 19;2021:8810074. doi: 10.1155/2021/8810074 (PMC8004372; doi:10.1155/2021/8810074)

100 bp M

C

+ve

1

2

3

4

NTC

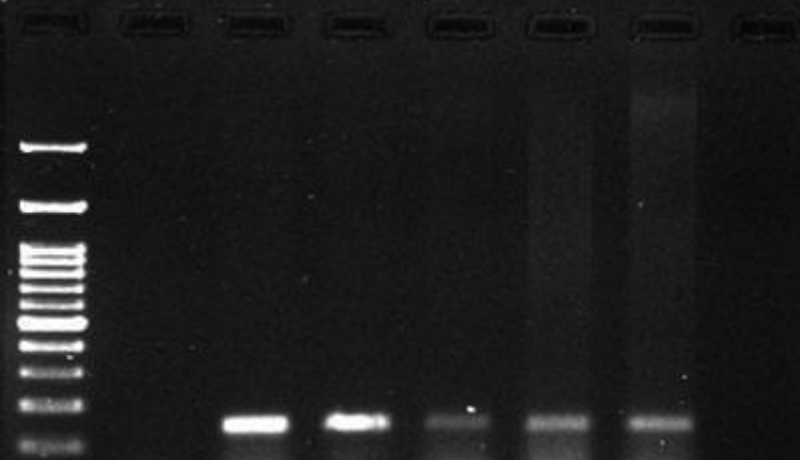

Supplement: Supplementary Materials — Figure S1: HPV DNA amplified by using Gp5+/Gp6 primers; lane 1 is 100 bp marker, lane 2 is control (normal sample), lane 3 is HPV-positive control, lanes 4-7 are patients' samples with a product length of 150 bp, and lane 8 is NTC (nontemplate control). [file 8810074.f1.pdf]
